# Report of the Special-purpose Committee on Names of Fungi with the Same Epithet, established at the XIX International Botanical Congress in Shenzhen, China

**DOI:** 10.1186/s43008-024-00150-z

**Published:** 2024-07-24

**Authors:** James K. Mitchell, David L. Hawksworth, Shaun R. Pennycook

**Affiliations:** 1 Farlow Herbarium and Library of Cryptogamic Botany, Harvard University, 22 Divinity Avenue, Cambridge, MA 02138 USA; 2 Comparative Fungal Biology, Royal Botanic Gardens, Kew, Surrey TW9 3AE UK; 3 Department of Life Sciences, The Natural History Museum, London, SW7 5BD UK; 4 Manaaki Whenua–Landcare Research, Private Bag 92170, Auckland, 1072 New Zealand

**Keywords:** Pleomorphic fungi, One fungus one name, 1F-1N

## Abstract

A Special-purpose Committee on Fungal Names with the Same Epithet was established at the XIX International Botanical Congress (IBC) in Shenzhen, China in 2017, with a mandate to report to the 12th International Mycological Congress (IMC) with recommendations on a preferred course of action with respect to names of pleomorphic fungi sharing the same epithet under the *International Code of Nomenclature for algae, fungi, and plants*. This report provides a synthesis of the deliberations from the Special-purpose Committee. We discuss the arguments for and against the proposed solution to the problems that have arisen regarding the nomenclature of fungi described in multiple morphs using the same epithet. We also propose a gentler method of addressing the problem using existing procedures.

## Background

Unlike many of the other organisms governed by the *International Code of Nomenclature for algae, fungi, and plants* (herein, the *Shenzhen Code*; Turland et al. 2018), fungi can frequently be found in multiple morphs, which were historically—and still can be—challenging to recognize as belonging to the same species. The various morphs of many species were described independently in different genera prior to the realization that these morphologically different "species" simply represented different morphs in the life cycle of the same fungus. After this realization there were some attempts to unify the nomenclature of the different morphs of the same species (e.g. Fuckel 1870) but the predominant practice was to continue naming different morphs—even when suspected or proved to belong to the same species—as separate species in different genera. This practice was eventually formalized by its inclusion in the nomenclatural *Codes* governing fungal names. The issue of which name was to be applied to a species including all its known morphs was addressed by declaring that names whose type included the meiosporic (sexual) morph had priority over ones in which the type represented the mitosporic (asexual) morph. This practice was convenient, if fundamentally ludicrous; authors with a single new pleomorphic species in hand were put in the position of having to describe two or more new "species" with different types, potentially in multiple new genera. To link these names in some way, many authors, especially those dealing with plant pathogens, adopted the practice of using the same epithet in the several new "species" names, or when describing a new morph of an already known fungus (provided they were not homonymous combinations). This practice was accepted as an option at the Stockholm Congress in 1950 (Lanjouw et al. 1952: Art. 69), and remained, with minor changes, in various editions of the *Code*.

These practices all came to an end at the Melbourne Congress when Article 59 was revised to end the separate naming of different morphs of the same fungal species from 1 January 2013 (McNeill et al. 2012). It allowed names of a species described based on any morph to compete on an equal footing according to the usual rules of priority, and explicitly disallowed the practice of describing a new taxon whose circumscription included the type of a legitimate published name based on another morph. This realignment of fungal nomenclature brought it more in line with the nomenclature of other taxa covered by the *Code*. The change has also allowed fungal nomenclature to adapt to modern sequence-based taxonomy which has much more frequently demonstrated—using molecular techniques—that morphs belong to the same species. Overall, the change has proved of great benefit to the mycological community. The concept of lists of protected names was introduced at the same Congress and expanded at the Shenzhen Congress in 2017 to deal with competing names of fungi (Turland et al. 2018: Art. F.2). One unfortunate consequence which remained to be addressed, however, was the well-established practice of using the same epithet for multiple heterotypic morph names of the same species; this meant that when applying Article 11.4 of the *Shenzhen Code*, adoption of an unfamiliar epithet could be required even when the name preventing use of the oldest epithet was described as a different morph of the name with the oldest epithet, with full recognition that they were conspecific at the time of publication.

This issue was first pointed out by Hawksworth et al. (2013), who informally proposed a solution to address the problem then, as did Hawksworth (2014). The problem and the proposed solution were discussed—along with other proposed changes to the *Code* affecting fungi—at the 10th International Mycological Congress (IMC) in Bangkok in August 2014. At this congress, a questionnaire was given to all congress participants to elicit opinions on several proposed changes to the *Code*, including that proposed by Hawksworth et al. (2013) and Hawksworth (2014). Of 84 delegates expressing a view on this issue, 86.9% supported the proposed solution (Redhead et al. 2014). As a result of this response, Hawksworth (2015) formally proposed changes to the *Code* (Prop. 085; see also Art. 59 Prop. A in Turland & Wiersema 2017). This proposal was presented to the Nomenclature Section of the XIX International Botanical Congress (IBC) in Shenzhen, China in 2017. The proposal was not supported by the Nomenclature Committee for Fungi, and the Nomenclature Section of that Congress referred it to a new Special-purpose Committee on Pleomorphic Fungi, with a mandate to report on the matter to the XX IBC (Turland and Wiersema 2017; Wilson 2019; Lindon et al. 2020). In addition, the creation of a Special-purpose Committee on Fungi named using the same Epithet for Asexual and Sexual States was proposed at the 11th IMC in San Juan in 2018, which since 2017 governs Chapter F of the *Code* (matters pertaining only to fungi; May et al. 2018). This proposal was accepted, and a Special-purpose Committee on Names of Fungi with the Same Epithet was established under the IMC to report to the 12th IMC in Maastricht, The Netherlands in 2024 (May et al. 2018; May et al. 2019). This Special-purpose Committee (SPC) is at present regarded as a renaming of the SPC on Pleomorphic Fungi set up in Shenzhen (May et al. 2018; Wilson 2019) and is reporting to the 12th IMC as the matter relates only to organisms treated as fungi.

The eight voting members of the Special-purpose Committee, as constituted, were David L. Hawksworth (U.K., Co-convener), Shaun Pennycook (New Zealand, Co-convener), James Mitchell (U.S.A., Secretary), Paul M. Kirk (U.K.), Konstanze Bensch (Germany), Amy Rossman (U.S.A.), John McNeill (U.K.) and Yi-Jian Yao (China). Tom May participated as a non-voting observer. Not all members as constituted took an active part in the deliberations of the Committee, and unfortunately illness precluded Yi-Jian Yao from contributing entirely.

A voluntary survey of Committee members' attitudes on Hawksworth's (2015) proposal on names of fungi with the same epithet was conducted early in the deliberations of the Committee. The attitudes of committee members who responded to the survey (*n* = 7) toward the proposal were well-balanced: 28.6% were in support, 28.6% were in opposition, 14.3% did not have a strong opinion either way, and 28.6% did not have a strong opinion either way but were inclined slightly to oppose the proposal.

## Names of fungi with the same epithet

The set of names at the heart of the deliberations of the committee, pairs (or triplets, etc.) of fungal basionyms sharing the same epithet and with all basionyms or the younger of the basionyms described explicitly as morphs of the same species (herein abbreviated as NFSE), fall into several categories:

1. NFSE with no additional heterotypic synonyms
2. NFSE with additional heterotypic synonyms
  2.1 Nomenclatural changes for the fungus in question are not of great concern
  2.2 Nomenclatural changes for the fungus in question are disadvantageous
    2.2.1 One or more additional heterotypic synonyms have priority over or are treated as having priority over all the NFSE
    2.2.2 All the NFSE have priority or are treated as having priority over all additional heterotypic synonyms
    2.2.3 One or more additional heterotypic synonyms have priority over or are treated as having priority over some, but not all, of the NFSE
      2.2.3.1 Desired current name is based on the names in the NFSE with priority over additional heterotypic synonyms as above
      2.2.3.2 Desired current name is based on one of the names in the NFSE threatened by an additional heterotypic synonym as above

The effects of applying the rules for determining the current name of a fungus in the *Shenzhen Code* to the various categories is summarized below:

1. The current name has the same epithet as the NFSE; no problem
2. The current name may have a different epithet from the NFSE; potential problem
  2.1 The current name may have a different epithet from the NFSE; no problem
  2.2 The current name may have a different epithet from the NFSE; potential problem
    2.2.1 The current name will have a different epithet from the NFSE; potential problem
    2.2.2 The current name will have the same epithet as the NFSE; no problem
    2.2.3 The current name may have a different epithet from the NFSE; potential problem
      2.2.3.1 The current name will have the same epithet as the NFSE; no problem
      2.2.3.2 The current name will have a different epithet from the NFSE; problem

As can be seen above, only two categories involve a problem. The first is category 2.2.1, where the current name will take the epithet from one of the heterotypic synonyms of the NFSE. Hawksworth's (2015) proposal would not address this problem, as conservation or protection would be needed to allow the NFSE to compete for priority. The second category is 2.2.3.2, where the current name will again definitely take the epithet from one of the heterotypic synonyms. In this case, Hawksworth's (2015) proposed solution would address this problem by allowing the threatened name to have priority from the older, unthreatened name from the NFSE.

We can provide an example of a name pair in category 2.2.3.2 from a medically relevant context which has not yet been the subject of a specific proposed solution. De Hoog & Smith (2004) proposed the combination *Magnusiomyces capitatus* (de Hoog, M.T. Sm. & E. Guého) de Hoog & M.T. Sm. based on *Dipodascus capitatus* de Hoog, M.T. Sm. & E. Guého, first published in de Hoog et al. 1986. This new "species" was originally proposed explicitly as the sexual morph of the asexual morph "species" *Geotrichum capitatum* (Diddens & Lodder) Arx based on the basionym *Trichosporon capitatum* Diddens & Lodder published in 1942 (de Hoog et al. 1986). Based on the synonymy presented by de Hoog & Smith (2004), the correct name for this species in the genus *Magnusiomyces* under the *Shenzhen Code* should be "*Magnusiomyces spicatus*" based on *Sporotrichum spicatum* Delitsch, published in 1943. Hawksworth's (2015) solution would treat *Dipodascus capitatus* as a combination based on *Trichosporon capitatum*, correcting it to "*Dipodascus capitatus* (Diddens & Lodder) de Hoog, M.T. Sm. & E. Guého," and make the correct name for this species "*Magnusiomyces capitatus* (Diddens & Lodder) de Hoog & M.T. Sm."

It would be convenient, and perhaps even crucial to have a complete list of those taxa falling into category 2.2.3.2. Unfortunately, the Special-purpose Committee has concluded that it is functionally impossible to generate a complete list of the NFSE. The two major fungal nomenclatural repositories, Index Fungorum and MycoBank, have retained some information on sexual-asexual morph connections; in examining records of such connections where the epithet of at least two morphs were the same, about 650 candidate NFSE were recovered from the Index Fungorum database and about 800 from the MycoBank database. This list is far from complete, as the practice of recording this information seems only to have begun with names published in the mid-1980s and ended in 2011. Presumably most names potentially presenting this pattern were therefore not recovered. In an additional complication the candidate NFSE recovered would have to have their protologues manually checked to verify that there was an explicit link made between the new morph name and that of the older, existing morph name(s) at the time of publication, allowing them to be considered NFSE at all. Apart from these problems, there is no automatic method for assessing the category each of the known candidate NFSE falls into: the existence of heterotypic synonyms and then whether the candidate NFSE is threatened by those heterotypic synonyms would have to be manually checked, a process complicated by the fact that the synonymies listed on the various available taxonomic databases (Species Fungorum, MycoBank, etc.) are variously up-to-date and frequently differ. The candidate NFSE would also need to be checked for frequency of usage by non-specialists to determine whether the fungus in question would be one for which nomenclatural changes would be disadvantageous. This exercise is also in some sense made futile by the fact that as taxonomic understandings and opinions change, the synonymy of names may change so that names currently in a "threatened" category move to a "safe" category and vice versa. The Special-purpose Committee can therefore not provide the number of total NFSE, nor the number of the NFSE which would be positively or negatively affected by Hawksworth's (2015) proposal. Many of the fungi involved, however, are plant pathogens or important in industry in food spoilage or medical mycology.

The deliberations of the Special-purpose Committee have ended in significant deadlock, with substantial arguments advanced for and against the adoption of the proposed changes. These arguments are presented below.

### In support of adopting Hawksworth's proposal

Adopting the proposed changes would alter the wording of Article F.8.1 to treat authors' valid descriptions of new "species" consisting of a new morph explicitly stated to be conspecific with a previously described legitimate species name and utilizing the same epithet as the previous name to be treated instead as a combination based on the previously published name. The proposed changes might desirably be altered so that any type associated with the new "species" name (now new combination) treated as a lectotype or neotype, if the requirement for such typifications are met, or as having no type status otherwise. This would probably not require any changes in wording to other parts of the *Code*, as the most relevant Articles already explicitly refer to Article F.8.1's exceptions. This approach has the benefit of being consistent with the spirit of many earlier authors who were merely describing another morph of a fungus they acknowledged as already being known; some were very uncomfortable with being forced to coin new names or even ignored the *Code*. The method also has the benefit of dealing with all possible problems at once, instead of waiting until new synonyms are discovered for NFSE and the problem arises. Action can be taken immediately by mycologists as such cases come to light without any need for the length of time it takes for proposals for conservation or protection to be made and decided. For example, the *Magnusiomyces capitatus* situation outlined above came up recently in the context of needing a “correct” name for use in a soon-to-be-published major reference work (de Hoog et al. 2023). Committee members supporting the proposal also consider that since any fungus in principle could eventually become one for which nomenclatural changes are disadvantageous, the proposed change could aid nomenclatural stability in the long-term as well as addressing the extant problems. The questionnaire circulated at IMC10 in Bangkok also had overwhelming support, as already mentioned, with 86.9% of respondents agreeing with it in principle (*n* = 84); it was similarly, though less overwhelmingly, supported when discussed by the International Committee for the Taxonomy of Fungi (67% in favor, *n* = 21; Redhead et al. 2014; Hawksworth 2015).

Committee members in support of the proposed changes suggest that failure to adopt them maintains a perverse situation where fungi were knowingly described multiple times with multiple distinct types. Doing nothing also leaves the burden of attempting to go through the slow and technical procedure for conservation or protection to researchers or doctors who frequently do not have much nomenclatural experience. Supporters also argue that to do nothing is to ignore an overwhelming desire for a solution to this problem in the mycological community, especially those dealing with economically important or disease-causing fungi.

### In opposition to adopting Hawksworth's proposal

Rejecting the proposed changes to the *Code* would allow the situation as it currently exists to play out. New morphs of existing species described as new species and satisfying the requirements for the description of a new species will continue to be treated as such. Committee members opposed to the proposed changes saw significant problems with them, which are outlined below.

A major criticism of the proposed changes is that, as currently worded, the proposed change does not seem to explicitly require that the author of the later morph explicitly cite the name of the earlier morph name the epithet they employ was taken from. Thus, the change extends an open-ended permission to designate as homotypic synonyms any two morphs with the same epithet, regardless of any other considerations. This is likely to be felt most keenly in the cases of names with host name-based epithets, which are common particularly in the older mycological literature. An additional minor criticism of the proposed change is that it does not apply to infraspecific taxa which can also be NFSEs. These criticisms could be most easily addressed by an alteration to the wording of the proposed change to make it clear that the link between the two morph names needs to be explicit, and that not only specific names are covered. As such, the problems are not fundamental, as are the remaining concerns.

The problem of disruption by the priority of unfamiliar synonyms that threaten established names is a commonplace of modern nomenclature, with new synonymies continually being uncovered by molecular analyses. It was felt that not enough sets NFSEs apart to warrant an exclusive special solution for them at the cost of a rejection of the type principle (Principle II: “The application of names of taxonomic groups is determined by means of nomenclatural types.”), one of the six fundamental principles of the *Code*. It was also felt that the proposed change represented an unwarranted major amendment to the *Code* to guard against largely hypothetical future problems when the remedy of proposals for conservation of protection is already available for these problems. The change was seen as particularly extreme since it does not fully protect the NFSEs. For instance, a nomenclatural change involving a change in epithet could be required for the names corrected by the proposed change to the *Code* if a previously unrecognized heterotypic synonym with priority were to be discovered; to maintain the same epithet in that case, a proposal for conservation, protection, or rejection would have to be drafted anyway if it was not already on one of the lists of protected names. The change also imposes disruptive nomenclatural changes in cases where they are neither desired nor necessary. For example, in many of the cases of NSFE pairs extracted from MycoBank, the accepted current name was neither member of the NFSE pair.

The unknown scale of the nomenclatural changes arising from the adoption of the proposed change could cause nomenclatural disruption far in excess of any gain to stability, not least since—without being able to enumerate the names affected beforehand—the nomenclatural databases would not be able to do a single update but would have to rely on piecemeal corrections over the course of years, undoubtedly causing confusion and perpetuating errors in the meantime. The urgency with which the proposal was made and mooted after the change in the *Melbourne Code* came into effect in 2013 was also perceived to have died down, with the most pressing cases the proposed change would have addressed having already been proposed for protection or conservation or otherwise addressed. At present relatively few names are known to exist for which this change would be helpful, and for which a more conventional solution has not already been pursued. It was felt that were a vote at IMC12 held with short arguments for and against the change presented in advance, support for the change could be lower than they were in 2014. An additional objection to this proposed change hinges on the fact that sometimes a new species described as a new morph of an earlier described species is later revealed to not be conspecific; many morph connections also remain unconfirmed by molecular methods. This is most critical in cases where the two morphs were described by different authors decades apart or based on material from different countries or continents or different hosts or substrates. An example of this is *Trichoderma tawa* P. Chaverri & Samuels, described in 2003 from Thailand on unknown bark as the asexual morph of *Hypocrea tawa* Dingley, described in 1952 from New Zealand on rotten wood of *Beilschmiedia tawa*; the connection between the two has since been rejected by Braithwaite et al. (2016) based on molecular analyses. It will be far easier, were the proposed changes implemented, for researchers to entirely neglect material associated with the now new combinations, a situation which could be widespread since NFSE pairs with no competing synonyms were the most numerous category (69/110) in a wide sampling of the NFSE data extracted from MycoBank. To relegate the descriptions of so many taxa to mere combinations will cause the loss of names of truly distinct fungi, necessitating new descriptions and potentially undesirable nomenclatural changes for them, as well as potentially causing the demoted type specimens representing these real and distinct taxa to be forgotten.

### An alternative possible solution

Rather than altering the *Code*, the situation could be remedied by creating a new list of protected names. This approach would circumvent the concerns voiced by those opposed to the proposed changes. It can also address several of the concerns of those in favor of the proposed changes. Submission of names to this list could be streamlined so that even workers with little nomenclatural expertise who notice a case where NSFEs cause a significant issue can easily submit the names for consideration without having to draft a formal proposal. Ideally, the turnaround time for considerations by the Nomenclature Committee for Fungi and General Committee would also be relatively short for approval of these names. This should be possible since there has now been a good amount of experience with this process since it was first implemented.

This solution would not address the concern over the fundamental problem that NFSEs represent a perverse situation where authors knowingly described as new the same species multiple times. However, this is equally true for many names of different morphs described as new with explicit citation of an earlier morph name where the later author did not choose to adopt the same epithet in the newer name. Since this is a situation that there has been no proposal to remedy, it may be that this concern could be tolerated in the case of NFSEs if the other concerns were satisfactorily addressed.

## Attitudes of the committee at the end of deliberations

A survey was conducted at the end of deliberations, with most participants not having changed their mind. Of those who participated in the final survey (*n* = 7), 42.9% supported the proposed changes to the *Code*, and 57.1% opposed the proposed change. The shift in opinion was due primarily to the three undecided Committee members taking more concrete stances.

A similar survey was conducted on the acceptability of a list of protected names with a proposed turnaround time for approval of less than a year. Of those who participated (*n* = 5), 60% approved of such a plan and 40% opposed it. The vote was split along the same lines as the previously mentioned vote, with at least one respondent casting doubt that a committee process could yield such a short turnaround time.

## Concluding remarks

The crux of the Committee's deliberations, and the thing that many members' opinions hinged on, was the number of cases that the proposed change to the *Code* would affect. While efforts to fully enumerate these cases by the Committee were stymied by the lack of systematic records, which would have made the search feasible, what could be gleaned from the records maintained by MycoBank and Index Fungorum allowed most people to make up their minds.

In the end, the Committee could not reach a consensus. Some members supported the proposed change as a common-sense fix to a problem created by an unfortunate historical practice, which was subsequently formalized. Other members favored employing already-existing methods for protection or conservation of these names (perhaps with additional streamlining), feeling the proposed change to be unnecessarily drastic for the scale of the problem. While supporting the rejection of the proposed change, these committee members were not in principle opposed to changing the *Code* to find a different, less drastic solution to this problem; they were merely unsure what such an alternative change to the *Code* could look like.
